# Supplementary material for: The Evolution and Characterization of the RNA Interference Pathways in Lophotrochozoa
Source: Genome Biol Evol. 2024 May 7;16(5):evae098. doi: 10.1093/gbe/evae098 (PMC11114477; doi:10.1093/gbe/evae098)
Supplement: evae098_Supplementary_Data [file evae098_supplementary_data.zip › Supplementary_materials.zip!/selected_miRNAs/NoGe_selected_miRNAs/mirdeep_pdf_files/NMRB01000064.1_Notospermus_geniculatus_strain_Ushimado_scaffold64,_whole_genome_shotgun_sequence_2361.pdf]

Provisional ID	:	NMRB01000064.1_Notospermus_geniculatus_strain_Ushimado_scaffold64,_whole_genome_shotgun_sequence_2361
Score total	:	48686.2
Score for star read(s)	:	3.9
Score for read counts	:	48681.3
Score for mfe	:	1.6
Score for randfold	:	0
Score for cons. seed	:	-0.6
Total read count	:	95498
Mature read count	:	75055
Loop read count	:	0
Star read count	:	20443

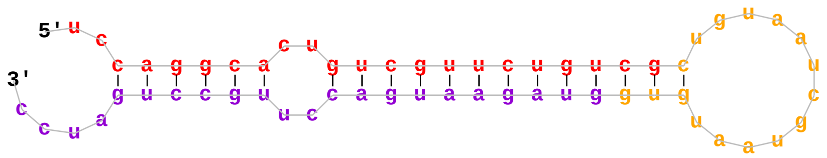

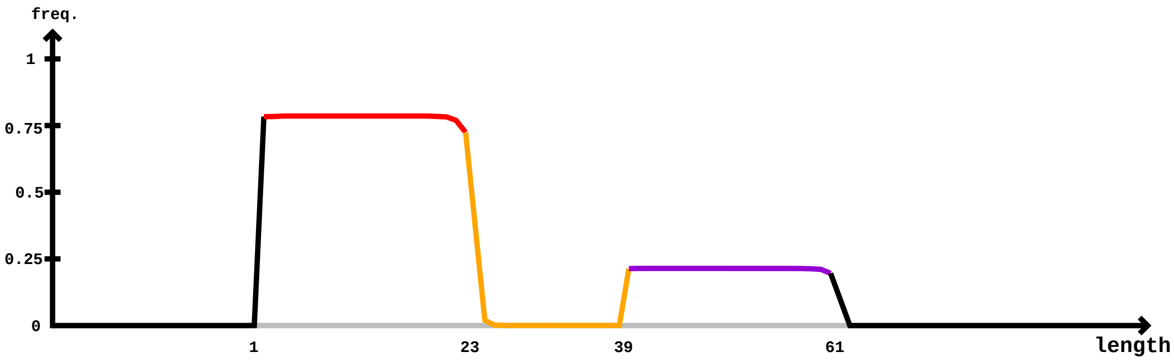

```
                  Mature                              Star
5'- ucauugacggacucgugggguuccaggcacugucguucugucgcuguaaucguaaugugguagaaugaccuugccugaucc ccaagggaacaucaacaucuggacacuugaac   -3'   obs
```

```
5'- ucauugacggacucguggguuccaggcacugucguucugucgcuguaaucguaaugugguagaaugaccuugccugaucccaagggaacaucaacaucuggacacuugaac   -3'    obs
    ucauugacggacucguggguuccaggcacugucguucugucgcuguaaucguaaugugguagaaugaccuugccugaucccaagggaacaucaacaucuggacacuugaac          exp
    ...((((.(..(((.((((...((((((..((((((((((((............))))))))))))..))))))...)))).)))..).)))).................    reads   mm   sample
    ...................Cuccaggcacugucguucugucg......................................................................    8       1    Fm3
    ....................uccaggcacugucAuucu..........................................................................    1       1    Fm3
    ....................uccaggcacugucguuAu..........................................................................    6       1    Fm3
    ....................uccaggcacugucguucu..........................................................................    63      0    Fm3
    ....................uccaggcacugucguucug.........................................................................    66      0    Fm3
    ....................uccaggcacugucguuAug.........................................................................    2       1    Fm3
    ....................uccaggcacugucguucuU.........................................................................    2       1    Fm3
    ....................ucAaggcacugucguucugu........................................................................    1       1    Fm3
    ....................uccaggcacugucguucGgu........................................................................    3       1    Fm3
    ....................uccaggcacugucguucugA........................................................................    6       1    Fm3
    ....................uccaggcacugucguucCgu........................................................................    3       1    Fm3
    ....................uccaggcacugucguuAugu........................................................................    3       1    Fm3
    ....................uccaggcacuCucguucugu........................................................................    2       1    Fm3
    ....................uccaggcacugucguucugu........................................................................    636     0    Fm3
    ....................uccaggcacugucguuGugu........................................................................    1       1    Fm3
    ....................uccaggcacuAucguucugu........................................................................    1       1    Fm3
    ....................uAcaggcacugucguucugu........................................................................    1       1    Fm3
    ....................Accaggcacugucguucugu........................................................................    1       1    Fm3
    ....................uccaggcacugucguuUugu........................................................................    2       1    Fm3
    ....................uccaggcacugucguucAgu........................................................................    6       1    Fm3
    ....................uccaggcacugGcguucuguc.......................................................................    1       1    Fm3
    ....................uccaggcacugucguucuguc.......................................................................    2107    0    Fm3
    ....................ucAaggcacugucguucuguc.......................................................................    3       1    Fm3
    ....................uccaggcacugucguucuAuc.......................................................................    2       1    Fm3
    ....................uccaggcaUugucguucuguc.......................................................................    10      1    Fm3
    ....................uccaggcacugucguucAguc.......................................................................    14      1    Fm3
    ....................Nccaggcacugucguucuguc.......................................................................    2       1    Fm3
    ....................ucGaggcacugucguucuguc.......................................................................    1       1    Fm3
    ....................uccaggcacugucguuAuguc.......................................................................    7       1    Fm3
    ....................uAcaggcacugucguucuguc.......................................................................    2       1    Fm3
    ....................Accaggcacugucguucuguc.......................................................................    1       1    Fm3
    ....................uccaggcacugucguuUuguc.......................................................................    4       1    Fm3
    ....................uccaUgcacugucguucuguc.......................................................................    1       1    Fm3
```

```
                    Mature                          Star
ucauugacggacucgugggucccaggcacugucguucugucgcuguaaucguaauguggguagaaugaccuugccugaucccaagggaacaucaacaucuggacacuugaac
....................uccaggcacugucguuGuguc.......................................................................    3      1    Fm3
....................uccaggcacugucguucGguc.......................................................................    8      1    Fm3
....................uccaggcacugucguucCguc.......................................................................    16     1    Fm3
....................uccaggcacuAucguucuguc.......................................................................    2      1    Fm3
....................uccaggcacuCucguucuguc.......................................................................    4      1    Fm3
....................uccaggcacugucguucuguU.......................................................................    47     1    Fm3
....................uccaggcacugucguucuguA.......................................................................    42     1    Fm3
....................uccaggcGcugucguucuguc.......................................................................    1      1    Fm3
....................ucUaggcacugucguucuguc.......................................................................    1      1    Fm3
....................uccaAgcacugucguucuguc.......................................................................    1      1    Fm3
....................uUcaggcacugucguucuguc.......................................................................    1      1    Fm3
....................uccGggcacugucguucuguc.......................................................................    2      1    Fm3
....................uccaggcacugucgAucuguc.......................................................................    1      1    Fm3
....................uccaggcacugucguucuguG.......................................................................    1      1    Fm3
....................uccaggcacuguUguucuguc.......................................................................    1      1    Fm3
....................uccaggcacuUucguucuguc.......................................................................    1      1    Fm3
....................Accaggcacugucguucugucg......................................................................    6      1    Fm3
....................uccaggcacugucguCcugucg......................................................................    7      1    Fm3
....................uccCggcacugucguucugucg......................................................................    2      1    Fm3
....................uccaggcacugucgCucugucg......................................................................    5      1    Fm3
....................uccaggcacugucgAucugucg......................................................................    2      1    Fm3
....................uccaggcacugucCuucugucg......................................................................    2      1    Fm3
....................uccaggcUcugucguucugucg......................................................................    7      1    Fm3
....................ucGaggcacugucguucugucg......................................................................    18     1    Fm3
....................Gccaggcacugucguucugucg......................................................................    1      1    Fm3
....................uccaggcacugCcguucugucg......................................................................    5      1    Fm3
....................uccaggcacugucUuucugucg......................................................................    5      1    Fm3
....................uccaggcacugucguucCgucg......................................................................    169    1    Fm3
....................uccaggcacugucguGcugucg......................................................................    2      1    Fm3
....................uccaggcacugucguAcugucg......................................................................    5      1    Fm3
....................uccaggcacugucguucugucC......................................................................    4      1    Fm3
....................Cccaggcacugucguucugucg......................................................................    9      1    Fm3
....................uccaggcacugucguuAugucg......................................................................    152    1    Fm3
....................uccaggcacCgucguucugucg......................................................................    4      1    Fm3
....................ucAaggcacugucguucugucg......................................................................    19     1    Fm3
....................uccagNcacugucguucugucg......................................................................    5      1    Fm3
....................uccaggcacugucguucAgucg......................................................................    166    1    Fm3
....................uccaggcacuCucguucugucg......................................................................    28     1    Fm3
....................uccaggcacuguUguucugucg......................................................................    12     1    Fm3
....................uUcaggcacugucguucugucg......................................................................    3      1    Fm3
....................uccaggcacugucguucugucg......................................................................    32238  0    Fm3
....................uccaggcacugucguucuCucg......................................................................    2      1    Fm3
....................uccaggcacugucguucugucU......................................................................    432    1    Fm3
....................uccaggcacGgucguucugucg......................................................................    3      1    Fm3
....................uccaggGacugucguucugucg......................................................................    3      1    Fm3
....................uccaggcacugucguucuguAg......................................................................    2      1    Fm3
....................uccaggcaAugucguucugucg......................................................................    1      1    Fm3
....................uccaggcacuAucguucugucg......................................................................    57     1    Fm3
....................uccaggcaUugucguucugucg......................................................................    9      1    Fm3
....................uAcaggcacugucguucugucg......................................................................    22     1    Fm3
....................uccaggAacugucguucugucg......................................................................    7      1    Fm3
....................uccUggcacugucguucugucg......................................................................    2      1    Fm3
....................Nccaggcacugucguucugucg......................................................................    17     1    Fm3
....................uccaggcacuguGguucugucg......................................................................    2      1    Fm3
....................uccaCgcacugucguucugucg......................................................................    2      1    Fm3
....................ucUaggcacugucguucugucg......................................................................    12     1    Fm3
....................uccaggcacuguAguucugucg......................................................................    7      1    Fm3
....................uccaggcacugucAuucugucg......................................................................    17     1    Fm3
....................uccaggcacugucguuUugucg......................................................................    61     1    Fm3
....................uccagUcacugucguucugucg......................................................................    3      1    Fm3
....................uccaggcacugucguucugucA......................................................................    71     1    Fm3
....................uccagCcacugucguucugucg......................................................................    8      1    Fm3
....................uccaggcacugucguucugAcg......................................................................    3      1    Fm3
....................uccaggUacugucguucugucg......................................................................    6      1    Fm3
....................uccaAgcacugucguucugucg......................................................................    5      1    Fm3
....................uccaggcacuUucguucugucg......................................................................    20     1    Fm3
....................uccaggcacugucguucGgucg......................................................................    158    1    Fm3
....................uccaUgcacugucguucugucg......................................................................    5      1    Fm3
....................uccaggcacugucguucugCcg......................................................................    2      1    Fm3
....................uGcaggcacugucguucugucg......................................................................    8      1    Fm3
```

```
                    Mature                                      Star
ucauugacggacucgugggu uccaggcacugucguucugucg cuguaaucguaaugug guagaaugaccuugccugaucc ccaagggaacaucaacaucuggacacuugaac
....................uccaggcacugAcguucugucg......................................................................  3     1  Fm3
....................uccaggcGcugucguucugucg......................................................................  2     1  Fm3
....................uccaggcacugucguucuAucg......................................................................  13    1  Fm3
....................uccaggcacugucguucuUucg......................................................................  9     1  Fm3
....................uccaggcacugucguuGugucg......................................................................  37    1  Fm3
....................uccaggcacugucguucuguGg......................................................................  2     1  Fm3
....................uccaggcacugucgGucugucg......................................................................  1     1  Fm3
....................uccGggcacugucguucugucg......................................................................  3     1  Fm3
....................uccaggNacugucguucugucg......................................................................  2     1  Fm3
....................uccagAcacugucguucugucg......................................................................  1     1  Fm3
....................uccaggcacugucguucuguUg......................................................................  7     1  Fm3
....................uccaggcacugucguucugucgG.....................................................................  4     1  Fm3
....................uccaggcacugucguucCgucgc.....................................................................  16    1  Fm3
....................uccaggcacugucguucugucAc.....................................................................  1     1  Fm3
....................uccaggcacugucguuUugucgc.....................................................................  1     1  Fm3
....................uccaggcacugucguucugucgA.....................................................................  1521  1  Fm3
....................uccaggcacugucguuAugucgc.....................................................................  10    1  Fm3
....................uccaggcacugucguucAgucgc.....................................................................  11    1  Fm3
....................uccaggcacugucguucugucUc.....................................................................  1     1  Fm3
....................uccaggcacuUucguucugucgc.....................................................................  1     1  Fm3
....................uccaggcacugucguuGugucgc.....................................................................  1     1  Fm3
....................uccUggcacugucguucugucgc.....................................................................  2     1  Fm3
....................Nccaggcacugucguucugucgc.....................................................................  2     1  Fm3
....................uccaggcacuCucguucugucgc.....................................................................  1     1  Fm3
....................uccaggcacuguGguucugucgc.....................................................................  1     1  Fm3
....................ucUaggcacugucguucugucgc.....................................................................  1     1  Fm3
....................uccaggcaUugucguucugucgc.....................................................................  3     1  Fm3
....................Accaggcacugucguucugucgc.....................................................................  1     1  Fm3
....................uccaggcacugucguucugucgc.....................................................................  1871  0  Fm3
....................uccaggcacGgucguucugucgc.....................................................................  1     1  Fm3
....................uccaggcacugucguucuguUgc.....................................................................  1     1  Fm3
....................uccaggcacugucAuucugucgc.....................................................................  2     1  Fm3
....................uccaggcacuAucguucugucgc.....................................................................  8     1  Fm3
....................Cccaggcacugucguucugucgc.....................................................................  1     1  Fm3
....................uccaggcacugucguucugucgU.....................................................................  897   1  Fm3
....................uccaggcacugucguucGgucgc.....................................................................  7     1  Fm3
....................uccaUgcacugucguucugucgc.....................................................................  1     1  Fm3
....................ucAaggcacugucguucugucgc.....................................................................  2     1  Fm3
....................uccagUcacugucguucugucgc.....................................................................  1     1  Fm3
....................uccaggcacuguAguucugucgc.....................................................................  1     1  Fm3
....................uccGggcacugucguucugucgc.....................................................................  1     1  Fm3
....................uccaggcacugucguuUugucgcu....................................................................  2     1  Fm3
....................uccaggcacugucguucugucgGu....................................................................  5     1  Fm3
....................uccaggcacugucguucAgucgcu....................................................................  1     1  Fm3
....................uccaggcacugucguucugucgcu....................................................................  251   0  Fm3
....................uccaggcacugucguucugucgcA....................................................................  168   1  Fm3
....................uccaggcacugucguucugucgUu....................................................................  341   1  Fm3
....................uccaggcacugucguucGgucgcu....................................................................  2     1  Fm3
....................uccaggcacugucguucugucgcG....................................................................  6     1  Fm3
....................uccaggcacugucguucugucgAu....................................................................  8     1  Fm3
....................uccaggcacugucguucCgucgcu....................................................................  3     1  Fm3
....................uccaggcacugucguucugucgAug...................................................................  7     1  Fm3
....................uccaggcacugucguucugucgcuA...................................................................  3     1  Fm3
....................uccaggcacugucguucugucgcuU...................................................................  16    1  Fm3
....................uccaggcacugucguucugucgcuUu..................................................................  1     1  Fm3
....................uccaggcacugucguucugucgcuguaauc..............................................................  1     0  Fm3
.....................Ucaggcacugucguucugucg......................................................................  3     1  Fm3
.....................ccaggcacugucguucugucgc.....................................................................  2     0  Fm3
.....................ccaggcacugucguucuCucgc.....................................................................  1     1  Fm3
.....................Ucaggcacugucguucugucgc.....................................................................  1     1  Fm3
.....................ccaggcacugucguucugucgcu....................................................................  6     0  Fm3
......................caggcacugucguucugu........................................................................  1     0  Fm3
......................caggcacugucguucuguc.......................................................................  6     0  Fm3
......................caggcacugucguuAugucg......................................................................  1     1  Fm3
......................caggcacugucCuucugucg......................................................................  1     1  Fm3
......................caggcacugucguucuguAg......................................................................  1     1  Fm3
......................caggcacugucguucugAcg......................................................................  1     1  Fm3
......................caggcacugucguucugGcg......................................................................  1     1  Fm3
......................caggcacugucguucugucg......................................................................  49    0  Fm3
......................caggcacugucguucugucU......................................................................  1     1  Fm3
```

Mature Star

```
ucauugacggacucgugggguuccaggcacugucguucugucgcuguaaucguaauguggguagaaugaccuugccugaucccaagggaacaucaacaucuggacacuugaac
......................caggcacugucguuUugucgc.....................................................................	1	1	Fm3
......................caggcacugucguucugucCc.....................................................................	1	1	Fm3
......................caggcacugucguucugucgc.....................................................................	4	0	Fm3
......................caggcacugucguucugucgU.....................................................................	2	1	Fm3
......................caggcacugucguucugCcgcu....................................................................	1	1	Fm3
......................caggcacugucguucugucgcu....................................................................	6	0	Fm3
......................caggcacugucguucugucgUu....................................................................	3	1	Fm3
......................caggcacugucguucuguAgcu....................................................................	1	1	Fm3
.......................aggcacugucguucuguc.......................................................................	5	0	Fm3
.......................Nggcacugucguucuguc.......................................................................	1	1	Fm3
..........................................cuguaaucguaauguggguagaaugaccuug........................................	1	0	Fm3
...........................................uguaaucguaauguggguagaaugaccuug........................................	9	0	Fm3
..........................................................guagaaugaccuugccug....................................	9	0	Fm3
..........................................................guagUaugaccuugccuga...................................	1	1	Fm3
..........................................................guagaaugaccuugccGga...................................	2	1	Fm3
..........................................................guagaaAgaccuugccuga...................................	1	1	Fm3
..........................................................guagaaugaccuugccuga...................................	63	0	Fm3
..........................................................guagaaugaccuugccugU...................................	4	1	Fm3
..........................................................guagaaugaccuugccuUa...................................	1	1	Fm3
..........................................................guagaaugacAuugccuga...................................	1	1	Fm3
..........................................................guagaaugacGuugccugau..................................	1	1	Fm3
..........................................................guagaaugaccuugccGgau..................................	1	1	Fm3
..........................................................guagaaugacUuugccugau..................................	1	1	Fm3
..........................................................guagaaugaccuugccugau..................................	95	0	Fm3
..........................................................guaAaaugaccuugccugau..................................	1	1	Fm3
..........................................................guagaaugaccuugccugaG..................................	1	1	Fm3
..........................................................guUgaaugaccuugccugau..................................	1	1	Fm3
..........................................................guagaaugacAuugccugau..................................	2	1	Fm3
..........................................................guagaaugGccuugccugau..................................	1	1	Fm3
..........................................................guagaaugaccuugccuUau..................................	2	1	Fm3
..........................................................guagaaCgaccuugccugau..................................	1	1	Fm3
..........................................................gGagaaugaccuugccugau..................................	1	1	Fm3
..........................................................guagaaugacAuugccugauc.................................	1	1	Fm3
..........................................................guagaaugaccuugccCgauc.................................	2	1	Fm3
..........................................................guagaUugaccuugccugauc.................................	1	1	Fm3
..........................................................guagaaugaccCugccugauc.................................	1	1	Fm3
..........................................................guagUaugaccuugccugauc.................................	1	1	Fm3
..........................................................guagaaugaccuuUccugauc.................................	1	1	Fm3
..........................................................guagaaugaccuugccAgauc.................................	2	1	Fm3
..........................................................guagaaugaccuuAccugauc.................................	2	1	Fm3
..........................................................guagaaugaccuugccGgauc.................................	3	1	Fm3
..........................................................guagaaugaccuugccugauU.................................	23	1	Fm3
..........................................................guagaaugaccuugccugauc.................................	567	0	Fm3
..........................................................guagaaugaccuugccuCauc.................................	2	1	Fm3
..........................................................guagaaugaccuugccugauA.................................	7	1	Fm3
..........................................................guagaaugaccuugccuUauc.................................	2	1	Fm3
..........................................................guagaaugaccuugccugaCc.................................	1	1	Fm3
..........................................................guagaaugaccuugccuAauc.................................	4	1	Fm3
..........................................................guaCaaugaccuugccugaucc................................	1	1	Fm3
..........................................................guagaaugaccuugccAgaucc................................	12	1	Fm3
..........................................................guagaaugaccuCgccugaucc................................	3	1	Fm3
..........................................................guagaaugaccuugccugaucU................................	121	1	Fm3
..........................................................guagaaugaccuugccGgaucc................................	7	1	Fm3
..........................................................guagGaugaccuugccugaucc................................	1	1	Fm3
..........................................................guagaaugaccuugccCgaucc................................	18	1	Fm3
..........................................................guagaaAgaccuugccugaucc................................	3	1	Fm3
..........................................................guagaaugaccuugccugaucA................................	8	1	Fm3
..........................................................guagaaugaccuugcAugaucc................................	2	1	Fm3
..........................................................guUgaaugaccuugccugaucc................................	1	1	Fm3
..........................................................guagaaugacAuugccugaucc................................	14	1	Fm3
..........................................................Uuagaaugaccuugccugaucc................................	2	1	Fm3
..........................................................guagaaugaccuugAcugaucc................................	3	1	Fm3
..........................................................guagaaugacUuugccugaucc................................	11	1	Fm3
..........................................................guagaaugaccuugccuCaucc................................	26	1	Fm3
..........................................................guagaaugaUcuugccugaucc................................	2	1	Fm3
..........................................................guagaaugaccuugccugauUc................................	7	1	Fm3
..........................................................guagaaugacGuugccugaucc................................	11	1	Fm3
..........................................................guagaUugaccuugccugaucc................................	1	1	Fm3
..........................................................guagaaugaccuugccugaAcc................................	1	1	Fm3
..........................................................guagaaugaccuGgccugaucc................................	1	1	Fm3
```

```
                   Mature                             Star
ucauugacggacucgugggiuuccaggcacugucguucugucgcuguaaucguaaugugguagaaugaccuugccugaucc
```

Mature **Star**

ucauugacggacucgugggu**uccaggcacugucguucugucg**cuguaaucguaaugug**guagaaugaccuugccugaucc**ccaagggaacaucaacaucuggacacuugaac			
....................ucUaggcacugucguucuguc...	1	1	Mm3
....................uccaggcacuCucguucuguc...	1	1	Mm3
....................uccaggcacugucguucuguA...	13	1	Mm3
....................uccaggcacugucgAucugucg..	1	1	Mm3
....................uccaggcacugucguucugucC..	2	1	Mm3
....................uccaggcacGgucguucugucg..	1	1	Mm3
....................Cccaggcacugucguucugucg..	3	1	Mm3
....................uccaggcacugucguucCgucg..	45	1	Mm3
....................uccaggcacugucguucGgucg..	37	1	Mm3
....................uccaggcacugucguucAgucg..	40	1	Mm3
....................uccagAcacugucguucugucg..	1	1	Mm3
....................uccaggUacugucguucugucg..	3	1	Mm3
....................uccaggcacugucgCucugucg..	1	1	Mm3
....................uccaggcacugucguuAugucg..	37	1	Mm3
....................uccaggcacuAucguucugucg..	15	1	Mm3
....................uGcaggcacugucguucugucg..	2	1	Mm3
....................uccaggcacugucguucuAucg..	1	1	Mm3
....................uccaggcacuguUguucugucg..	5	1	Mm3
....................uccaggcacuCucguucugucg..	8	1	Mm3
....................uccaggcacugucguucugCcg..	3	1	Mm3
....................uccaggcacugAcguucugucg..	2	1	Mm3
....................ucUaggcacugucguucugucg..	2	1	Mm3
....................uccaggcacugucguucugucg..	7405	0	Mm3
....................uccaUgcacugucguucugucg..	2	1	Mm3
....................uccagUcacugucguucugucg..	2	1	Mm3
....................uccaggcacugucCuucugucg..	1	1	Mm3
....................uccaggcacugucguucuguUg..	1	1	Mm3
....................uUcaggcacugucguucugucg..	3	1	Mm3
....................uAcaggcacugucguucugucg..	6	1	Mm3
....................uccagNcacugucguucugucg..	1	1	Mm3
....................uccaggcacugucguucugucA..	42	1	Mm3
....................uccaggcacuUucguucugucg..	6	1	Mm3
....................ucAaggcacugucguucugucg..	1	1	Mm3
....................uccaggcacugucguucugucU..	112	1	Mm3
....................uccaggcacugucguuUugucg..	14	1	Mm3
....................uccGggcacugucguucugucg..	3	1	Mm3
....................uccaggcacugucguuGugucg..	7	1	Mm3
....................uccaggcacugucguCcugucg..	1	1	Mm3
....................uccaggcUcugucguucugucg..	1	1	Mm3
....................Nccaggcacugucguucugucg..	2	1	Mm3
....................uccaggcacugucAuucugucg..	2	1	Mm3
....................uccaggcacCgucguucugucg..	1	1	Mm3
....................uccaggcacugucguucugGcg..	1	1	Mm3
....................uccaggcacugucgGucugucg..	2	1	Mm3
....................uccaggcacugucguucugAcg..	1	1	Mm3
....................uccaggcacugucguucugucgA...	274	1	Mm3
....................uccaggcacugucguucGgucgc...	6	1	Mm3
....................uccaggcUcugucguucugucgc...	1	1	Mm3
....................uccaggcacugucguucAgucgc...	7	1	Mm3
....................uccaggcacugucguucugucgU...	250	1	Mm3
....................uccaggcacugucguuAugucgc...	1	1	Mm3
....................uccaggcacuAucguucugucgc...	1	1	Mm3
....................uccaggcacugucguucCgucgc...	2	1	Mm3
....................uccaggcacugucguucugucgG...	8	1	Mm3
....................uAcaggcacugucguucugucgc...	1	1	Mm3
....................uccaggNacugucguucugucgc...	1	1	Mm3
....................uccaggcacuUucguucugucgc...	1	1	Mm3
....................uccaggcacugucgCucugucgc...	1	1	Mm3
....................uccaggcacuCucguucugucgc...	2	1	Mm3
....................uccaggcacugucguucugucgc...	561	0	Mm3
....................uccaCgcacugucguucugucgc...	1	1	Mm3
....................uccCggcacugucguucugucgc...	1	1	Mm3
....................Nccaggcacugucguucugucgc...	1	1	Mm3
....................uccCggcacugucguucugucgcu..	1	1	Mm3
....................uccaggcacugucguucGgucgcu..	1	1	Mm3
....................uccaggcacugucguucugucgcA..	41	1	Mm3
....................uccaggcacugucguucugucgcG..	5	1	Mm3
....................uccaggcacugucguucugucgUu..	89	1	Mm3
....................uccaggcacugucguucugucgcC..	2	1	Mm3
....................uccaggcacugucguucAgucgcu..	1	1	Mm3

```
                    Mature                                      Star
ucauugacggacucgugggguuccaggcacugucguucugucgcuguaaucguaauguggguagaaugaccuugccugaucccaagggaacaucaacaucuggacacuugaac
```

```
....................uccaggcacugucguucCgucgcu....................................................................    1     1   Mm3
....................uccaggcacugucguucugucgAu....................................................................    4     1   Mm3
....................uccaggcacugucguucugucgcu....................................................................    129   0   Mm3
....................uccaggcacugucguuGugucgcu....................................................................    2     1   Mm3
....................uccaggcacugucguucugucgcuU...................................................................    12    1   Mm3
....................uccaggcacugucguucugucgcuUu..................................................................    1     1   Mm3
....................uccaggcacugucguucugucgcuAu..................................................................    1     1   Mm3
....................uccaggcacugucguucGgucgcuguaauc..............................................................    1     1   Mm3
....................uccaggcacugucguucugucgcuguaauc..............................................................    7     0   Mm3
.....................ccaggcacugucguucugucg......................................................................    2     0   Mm3
.....................ccaggcacugucguucugucgc.....................................................................    2     0   Mm3
......................caggcacugucguucuguU.......................................................................    1     1   Mm3
......................caggcacugucguucugucg......................................................................    22    0   Mm3
......................cagCcacugucguucugucg......................................................................    1     1   Mm3
......................caggcacugucguucugCcg......................................................................    1     1   Mm3
......................caggcacugucguucugucgA.....................................................................    1     1   Mm3
......................caggcacugucguucugucgc.....................................................................    5     0   Mm3
......................caggcacugucguucugucgU.....................................................................    1     1   Mm3
......................caggcacugucguucugucgUu....................................................................    1     1   Mm3
.......................aggcacugucguucuguc.......................................................................    1     0   Mm3
.......................aggcacugucguucugucg......................................................................    1     0   Mm3
.......................aggcacugucguucugucgcu....................................................................    2     0   Mm3
..........................................cuguaaucguaaugugguagaaugaccu..........................................    1     0   Mm3
...........................................uguaaucguaaugugguagaaugaccuugcc......................................    1     0   Mm3
...........................................uguaaucguaaugugguagaaugaccuugccugaucc................................    2     0   Mm3
..........................................................guagaaugaccuugccug....................................    5     0   Mm3
..........................................................guagaaugaccuugccuga...................................    5     0   Mm3
..........................................................guagaaugaccuugccugU...................................    1     1   Mm3
..........................................................guagaaugaccuugccuAau..................................    1     1   Mm3
..........................................................guagaaugaccuugccugau..................................    18    0   Mm3
..........................................................guagaaugacAuugccugauc.................................    1     1   Mm3
..........................................................guagaaugaccuugccugauc.................................    110   0   Mm3
..........................................................guagaaugaccuugccugauU.................................    2     1   Mm3
..........................................................guagaaCgaccuugccugauc.................................    1     1   Mm3
..........................................................guagaaugaccuugccugauA.................................    1     1   Mm3
..........................................................guagaaugUccuugccugaucc................................    1     1   Mm3
..........................................................guagaaugaccuugccuUaucc................................    6     1   Mm3
..........................................................Nuagaaugaccuugccugaucc................................    1     1   Mm3
..........................................................guagaauUaccuugccugaucc................................    1     1   Mm3
..........................................................guagaaugaccuugccugGucc................................    1     1   Mm3
..........................................................guagaaugaccuugccugaucc................................    1709  0   Mm3
..........................................................guagGaugaccuugccugaucc................................    1     1   Mm3
..........................................................guagaaAgaccuugccugaucc................................    1     1   Mm3
..........................................................guagaaugaccCugccugaucc................................    1     1   Mm3
..........................................................gCagaaugaccuugccugaucc................................    1     1   Mm3
..........................................................guGgaaugaccuugccugaucc................................    1     1   Mm3
..........................................................guagaaugacUuugccugaucc................................    1     1   Mm3
..........................................................guagaaugacAuugccugaucc................................    2     1   Mm3
..........................................................Uuagaaugaccuugccugaucc................................    2     1   Mm3
..........................................................guagaaugaccuugccugaucU................................    22    1   Mm3
..........................................................guagaaugaccuugccugaucA................................    1     1   Mm3
..........................................................guagaaNgaccuugccugaucc................................    1     1   Mm3
..........................................................guaAaaugaccuugccugaucc................................    1     1   Mm3
..........................................................guagaaugaccuugccCgaucc................................    5     1   Mm3
..........................................................guagCaugaccuugccugaucc................................    3     1   Mm3
..........................................................guagUaugaccuugccugaucc................................    2     1   Mm3
..........................................................guagaaugaccuugUcugaucc................................    1     1   Mm3
..........................................................guagaaugaccuugccuCaucc................................    11    1   Mm3
..........................................................guagaaugacGuugccugaucc................................    2     1   Mm3
..........................................................guagaaugaccuugccAgaucc................................    2     1   Mm3
..........................................................guagaaugaccuugccuAaucc................................    5     1   Mm3
..........................................................guagaaugaccuugccugauAc................................    2     1   Mm3
..........................................................guagaaugaccuugccugauccA...............................    92    1   Mm3
..........................................................guagaaugaccuugccugauccU...............................    387   1   Mm3
..........................................................guagaaugaccuugccugauccG...............................    8     1   Mm3
..........................................................guagaaugaccuugccugauccc...............................    2     0   Mm3
...........................................................uagaaugaccuugccugaucc................................    3     0   Mm3
...........................................................uagaaugaccuugccugauccU...............................    3     1   Mm3

...................Cuccaggcacugucguucuguc.......................................................................    3     1   Fm2
```

```
                    Mature                                      Star
ucauugacggacucgugggucccaggcacugucguucugucgcuguaaucguaaugugguagaaugaccuugccugaucccaagggaacaucaacaucuggacacuugaac
```

```
ucauugacggacucgugggu uccaggcacugucguucugucg cuguaaucguaaugug guagaaugaccuugccugaucc ccaagggaacaucaacaucuggacacuugaac
....................uccaggcacugucguucu..........................................................................	2	0	Fm2
....................uccaggcacugucguucug.........................................................................	8	0	Fm2
....................uccaggcacugucguucAgu........................................................................	2	1	Fm2
....................uccaggcacugucguucugu........................................................................	26	0	Fm2
....................uccaggcacugucguucugG........................................................................	3	1	Fm2
....................uccaggcacugucguucuguU.......................................................................	10	1	Fm2
....................uccaggcacugucguucuUuc.......................................................................	1	1	Fm2
....................uccaggcacugucguucuguc.......................................................................	121	0	Fm2
....................uccaggcacugucguucuguA.......................................................................	10	1	Fm2
....................uccaggcacugucguucugGcg......................................................................	1	1	Fm2
....................uccaggcacugucguuGugucg......................................................................	2	1	Fm2
....................uccaggcacugucguucGgucg......................................................................	5	1	Fm2
....................uccaggcacugucguuAugucg......................................................................	3	1	Fm2
....................uGcaggcacugucguucugucg......................................................................	1	1	Fm2
....................uccaggcacugucguucugucU......................................................................	2	1	Fm2
....................uccGggcacugucguucugucg......................................................................	1	1	Fm2
....................ucAaggcacugucguucugucg......................................................................	1	1	Fm2
....................uUcaggcacugucguucugucg......................................................................	1	1	Fm2
....................uccagUcacugucguucugucg......................................................................	1	1	Fm2
....................Nccaggcacugucguucugucg......................................................................	2	1	Fm2
....................uccaggcacugucgCucugucg......................................................................	1	1	Fm2
....................uccaggcacugucguucugucA......................................................................	6	1	Fm2
....................uccaggcacugucguucuguAg......................................................................	1	1	Fm2
....................uccaggcUcugucguucugucg......................................................................	1	1	Fm2
....................uccaggcacugucAuucugucg......................................................................	1	1	Fm2
....................uccaggcacugucguucugucg......................................................................	1102	0	Fm2
....................uccaggcacugucguucAgucg......................................................................	8	1	Fm2
....................uccaggcacugucguucuUucg......................................................................	1	1	Fm2
....................uccaggcacuUucguucugucg......................................................................	2	1	Fm2
....................ucGaggcacugucguucugucg......................................................................	1	1	Fm2
....................ucUaggcacugucguucugucg......................................................................	1	1	Fm2
....................uccaggcacuAucguucugucg......................................................................	2	1	Fm2
....................uccaggcacugucguuUugucg......................................................................	1	1	Fm2
....................uccaggcacugucguucCgucg......................................................................	6	1	Fm2
....................uccaggcacugucguucugucgU.....................................................................	31	1	Fm2
....................uccaggcacugucguucCgucgc.....................................................................	1	1	Fm2
....................uccaggcacugucguucugucgc.....................................................................	117	0	Fm2
....................uccaggcacugucguucAgucgc.....................................................................	1	1	Fm2
....................uccaggcacugucguucGgucgc.....................................................................	1	1	Fm2
....................uccaggcacugucguucugucgG.....................................................................	2	1	Fm2
....................uccaggcacugucguucugucgA.....................................................................	83	1	Fm2
....................uccaggcacugucguucugucgcA....................................................................	9	1	Fm2
....................uccaggcacugucguucugucgcG....................................................................	7	1	Fm2
....................uccaggcacugucguucugucgUu....................................................................	13	1	Fm2
....................uccaggcacugucguucugucgcu....................................................................	91	0	Fm2
....................uccaggcacugucguucugucgcuU...................................................................	18	1	Fm2
......................caggcacugucguucugucg......................................................................	9	0	Fm2
......................caggcacugucguAcugucg......................................................................	1	1	Fm2
..........................................................guagaaugaccuugccugau..................................	5	0	Fm2
..........................................................guagaaugaccuugccugCuc.................................	1	1	Fm2
..........................................................guagaaugaccuugccugauc.................................	177	0	Fm2
..........................................................guagaaugaccuugccugauU.................................	2	1	Fm2
..........................................................Nuagaaugaccuugccugauc.................................	1	1	Fm2
..........................................................guagaaugacAuugccugauc.................................	1	1	Fm2
..........................................................guagaaugaccuugcAugauc.................................	1	1	Fm2
..........................................................guagaaugaccuugccuAauc.................................	2	1	Fm2
..........................................................guagaaugaccuugccugUucc................................	1	1	Fm2
..........................................................guagaaugaccuugccGgaucc................................	2	1	Fm2
..........................................................guagaaugaccuugAcugaucc................................	1	1	Fm2
..........................................................guagUaugaccuugccugaucc................................	1	1	Fm2
..........................................................guagaaCgaccuugccugaucc................................	1	1	Fm2
..........................................................guagaaugaccuugccugaAcc................................	2	1	Fm2
..........................................................guagaUugaccuugccugaucc................................	1	1	Fm2
..........................................................guagaaugaccuugccugaucc................................	1296	0	Fm2
..........................................................guagaaugaccuugccuAaucc................................	3	1	Fm2
..........................................................guagaaugGccuugccugaucc................................	1	1	Fm2
..........................................................guagaaugaccuugccuCaucc................................	6	1	Fm2
..........................................................guagaaugaccuugccCgaucc................................	3	1	Fm2
..........................................................guagaaugaccuugccugaucU................................	27	1	Fm2
..........................................................guagaauCaccuugccugaucc................................	1	1	Fm2
```

```
                    Mature                              Star
ucauugacggacucgugggruccaggcacugucguucugucgcuguaaucguaaugugguagaaugaccuugccugaucccaagggaacaucaacaucuggacacuugaac
..........................................................guagaaugaccuugccAgaucc................................  2      1   Fm2
..........................................................guagGaugaccuugccugaucc................................  1      1   Fm2
..........................................................guagaaugaAcuugccugaucc................................  2      1   Fm2
..........................................................guagaaugaccuugUcugaucc................................  1      1   Fm2
..........................................................guagaaugaccuugccugauAc................................  1      1   Fm2
..........................................................guagaaugaccuugccuUaucc................................  7      1   Fm2
..........................................................guagaaugacAuugccugaucc................................  3      1   Fm2
..........................................................guagaaugacUuugccugaucc................................  3      1   Fm2
..........................................................guUgaaugaccuugccugaucc................................  1      1   Fm2
..........................................................guagaauAaccuugccugaucc................................  1      1   Fm2
..........................................................guagaaugacGuugccugaucc................................  2      1   Fm2
..........................................................guagaaugaccuugccugauccG...............................  9      1   Fm2
..........................................................guagaaugaccuugccugauccU...............................  114    1   Fm2
..........................................................guagaaugaccuugccugauccA...............................  45     1   Fm2

....................uccaggcacugucguucu..........................................................................  12     0   Fm1
....................uccaggcacugucguucug.........................................................................  10     0   Fm1
....................uccaggcacugucguucugA........................................................................  2      1   Fm1
....................uccaggcacugucguucugu........................................................................  23     0   Fm1
....................uccGggcacugucguucuguc.......................................................................  1      1   Fm1
....................uccaggcacugucguucuguU.......................................................................  3      1   Fm1
....................uccaggcacuCucguucuguc.......................................................................  1      1   Fm1
....................uccaggcacugucguucuguc.......................................................................  127    0   Fm1
....................uccaggcacugucguucGguc.......................................................................  1      1   Fm1
....................uccaggcacugucguucAguc.......................................................................  1      1   Fm1
....................uccaggcacugucguucuguA.......................................................................  1      1   Fm1
....................uccaggcacugucguucugucA......................................................................  5      1   Fm1
....................uccGggcacugucguucugucg......................................................................  1      1   Fm1
....................uccaggcacugucguucugucU......................................................................  20     1   Fm1
....................uUcaggcacugucguucugucg......................................................................  1      1   Fm1
....................uccaggcacugucguucugucg......................................................................  2119   0   Fm1
....................uccaggcacAgucguucugucg......................................................................  1      1   Fm1
....................uccaggcacugucguuUugucg......................................................................  5      1   Fm1
....................ucUaggcacugucguucugucg......................................................................  5      1   Fm1
....................uccagNcacugucguucugucg......................................................................  1      1   Fm1
....................uccaggcacuUucguucugucg......................................................................  3      1   Fm1
....................uccaUgcacugucguucugucg......................................................................  1      1   Fm1
....................uccaggcacugucguuGugucg......................................................................  10     1   Fm1
....................uccaggcacuAucguucugucg......................................................................  6      1   Fm1
....................uccaggcacugucguucAgucg......................................................................  12     1   Fm1
....................ucGaggcacugucguucugucg......................................................................  2      1   Fm1
....................uccaggcacuCucguucugucg......................................................................  2      1   Fm1
....................uccaggcacugucAuucugucg......................................................................  1      1   Fm1
....................uccaggcUcugucguucugucg......................................................................  1      1   Fm1
....................uccaAgcacugucguucugucg......................................................................  1      1   Fm1
....................uccaggcacugucguucCgucg......................................................................  17     1   Fm1
....................ucAaggcacugucguucugucg......................................................................  1      1   Fm1
....................uccaggcacugucguuAugucg......................................................................  3      1   Fm1
....................uccaggcacugucguucGgucg......................................................................  9      1   Fm1
....................uccaggcacugucguucugucgU.....................................................................  51     1   Fm1
....................uccaggcacugucguuAugucgc.....................................................................  1      1   Fm1
....................uccaggcacugucguucAgucgc.....................................................................  1      1   Fm1
....................uccaggcacugucguucugucgG.....................................................................  4      1   Fm1
....................uccaggcacugucguucugucgA.....................................................................  26     1   Fm1
....................uccaggcacugucguucCgucgc.....................................................................  1      1   Fm1
....................uccaggcacugucguucGgucgc.....................................................................  1      1   Fm1
....................uAcaggcacugucguucugucgc.....................................................................  1      1   Fm1
....................uccaggcacugucguuUugucgc.....................................................................  1      1   Fm1
....................uccaggcacugucguucugucgc.....................................................................  195    0   Fm1
....................uccaggcacugucguucugucgcG....................................................................  1      1   Fm1
....................uccaggcacugucguucugucgcA....................................................................  6      1   Fm1
....................Nccaggcacugucguucugucgcu....................................................................  1      1   Fm1
....................uccaggcacugucguucCgucgcu....................................................................  1      1   Fm1
....................uccaggcacugucguucugucgcu....................................................................  17     0   Fm1
....................uccaggcacugucguucugucgUu....................................................................  5      1   Fm1
....................uccaggcacugucguucugucgcug...................................................................  1      0   Fm1
....................uccaggcacugucguucugucgcuU...................................................................  6      1   Fm1
.....................ccaggcacugucguucugucgc.....................................................................  5      0   Fm1
......................caggcacugucguucugucg......................................................................  1      0   Fm1
..........................................cuguaaucguaaugugguagaaugaccuugccugaucc................................  1      0   Fm1
```

Mature **Star**

```
ucauugacggacucgugggucccaggcacugucguucugucgcuguaaucguaaugugguagaaugaccuugccugaucc ccaagggaacaucaacaucuggacacuugaac
```

```
ucauugacggacucgugggu uccaggcacugucguucugucg cuguaaucguaaugug guagaaugaccuugccugaucc ccaagggaacaucaacaucuggacacuugaac
...........................................uguaaucguaaugugguagaaugaccuugccugauc.................................  1     0  Fm1
..........................................................guagaaugaccuugccuga...................................  1     0  Fm1
..........................................................guagaaugaccuugccugaA..................................  1     1  Fm1
..........................................................guagaaugaccuugccugau..................................  27    0  Fm1
..........................................................guagaaugaccuugccugUu..................................  1     1  Fm1
..........................................................guagaaugacGuugccugau..................................  1     1  Fm1
..........................................................guagaaugaccuugccuCau..................................  1     1  Fm1
..........................................................guagaaugacAuugccugau..................................  1     1  Fm1
..........................................................guagaaugaccCugccugauc.................................  1     1  Fm1
..........................................................guagaaugaccuugccCgauc.................................  2     1  Fm1
..........................................................guagaaugaccuugccugauc.................................  166   0  Fm1
..........................................................Uuagaaugaccuugccugauc.................................  1     1  Fm1
..........................................................guagaaugaccuugccugauU.................................  3     1  Fm1
..........................................................guagaaugaccuugccuAauc.................................  5     1  Fm1
..........................................................guagaGugaccuugccugaucc................................  2     1  Fm1
..........................................................guagaaugaccuugccugaucA................................  2     1  Fm1
..........................................................guagaaugaccuugccugUucc................................  1     1  Fm1
..........................................................guagaaCgaccuugccugaucc................................  1     1  Fm1
..........................................................guagaaugaccuugccugaAcc................................  2     1  Fm1
..........................................................guagaaugaccuugccugaucc................................  1431  0  Fm1
..........................................................guagaaugaccuugccugaucU................................  39    1  Fm1
..........................................................guagGaugaccuugccugaucc................................  1     1  Fm1
..........................................................guagaaugUccuugccugaucc................................  2     1  Fm1
..........................................................guagaauUaccuugccugaucc................................  2     1  Fm1
..........................................................guagaaugacUuugccugaucc................................  2     1  Fm1
..........................................................guagaaugaccuGgccugaucc................................  1     1  Fm1
..........................................................guagaauAaccuugccugaucc................................  2     1  Fm1
..........................................................guGgaaugaccuugccugaucc................................  1     1  Fm1
..........................................................guagaauCaccuugccugaucc................................  1     1  Fm1
..........................................................guagaaugaccuugccGgaucc................................  2     1  Fm1
..........................................................guagaaugacAuugccugaucc................................  3     1  Fm1
..........................................................guagaaugaccuugccCgaucc................................  6     1  Fm1
..........................................................guagaaugaccuugccugaCcc................................  1     1  Fm1
..........................................................guagaaugaccuugccuCaucc................................  2     1  Fm1
..........................................................guagaaugaccuugccAgaucc................................  9     1  Fm1
..........................................................guagaaugaccuugccuAaucc................................  7     1  Fm1
..........................................................guagaaugacGuugccugaucc................................  2     1  Fm1
..........................................................guagaaugaccuugccuUaucc................................  2     1  Fm1
..........................................................guagaaugaccCugccugaucc................................  2     1  Fm1
..........................................................guagaaugaccuugccugauccG...............................  31    1  Fm1
..........................................................guagaaugaccuugccugauccU...............................  508   1  Fm1
..........................................................guagaaugaccuugccugauccc...............................  2     0  Fm1
..........................................................guagaaugaccuugccugauccA...............................  125   1  Fm1
.............................................................gaaugaccuugccugaucc................................  3     0  Fm1

...................Cuccaggcacugucguucugucg......................................................................  8     1  Mm1
....................uccaggcacugucguucu..........................................................................  5     0  Mm1
....................uccaggcacugucguucug.........................................................................  16    0  Mm1
....................uccaggcacugucguucCgu........................................................................  2     1  Mm1
....................uccaggcacugucguucGgu........................................................................  1     1  Mm1
....................uccaggcacugucguucAgu........................................................................  2     1  Mm1
....................uccaggcacugucguucugu........................................................................  152   0  Mm1
....................uccaggcacugucguucugG........................................................................  1     1  Mm1
....................uccaggcacugucguCcuguc.......................................................................  1     1  Mm1
....................uccaggcacugucguuAuguc.......................................................................  2     1  Mm1
....................uccaggcacugucguucGguc.......................................................................  2     1  Mm1
....................uccaggcacugucguucCguc.......................................................................  3     1  Mm1
....................uccaggcacugucguucAguc.......................................................................  4     1  Mm1
....................uccaggcacugucguucuguc.......................................................................  479   0  Mm1
....................uccaggcacugucguAcugucg......................................................................  1     1  Mm1
....................uAcaggcacugucguucugucg......................................................................  4     1  Mm1
....................uccaAgcacugucguucugucg......................................................................  1     1  Mm1
....................uccaggcacugucAuucugucg......................................................................  1     1  Mm1
....................uccaggcacuUucguucugucg......................................................................  4     1  Mm1
....................uccaggcacugucguucuCucg......................................................................  1     1  Mm1
....................uccUggcacugucguucugucg......................................................................  3     1  Mm1
....................uccaggcacugucguuGugucg......................................................................  12    1  Mm1
....................uccaggcacugucguucuguAg......................................................................  1     1  Mm1
....................uccaggcacuAucguucugucg......................................................................  15    1  Mm1
....................Nccaggcacugucguucugucg......................................................................  3     1  Mm1
```

```
                    Mature                                Star
ucauugacggacucguggguuccaggcacugucguucugucgcuguaaucguaauguggguagaaugaccuugccugaucccaagggaacaucaacaucuggacacuugaac
....................uccaggcacugucguucugucA......................................................................   9      1   Mm1
....................uccagNcacugucguucugucg......................................................................   2      1   Mm1
....................uccaggcacugucguucugucg......................................................................   6904   0   Mm1
....................uccaggcacugucgCucugucg......................................................................   2      1   Mm1
....................uccaggcacAgucguucugucg......................................................................   1      1   Mm1
....................uccaggcacugucguucGgucg......................................................................   36     1   Mm1
....................Accaggcacugucguucugucg......................................................................   2      1   Mm1
....................uccaggcUcugucguucugucg......................................................................   2      1   Mm1
....................uccaggcacugucguucuguUg......................................................................   1      1   Mm1
....................uccaggcacugucguucugucU......................................................................   86     1   Mm1
....................uccagUcacugucguucugucg......................................................................   3      1   Mm1
....................uccaggcacuguUguucugucg......................................................................   1      1   Mm1
....................uccaggcacugucguuAugucg......................................................................   29     1   Mm1
....................uccaggcacugucguucuAucg......................................................................   2      1   Mm1
....................uccaCgcacugucguucugucg......................................................................   1      1   Mm1
....................ucUaggcacugucguucugucg......................................................................   1      1   Mm1
....................uccaggcacugCcguucugucg......................................................................   2      1   Mm1
....................uccaggcacugucguucugAcg......................................................................   2      1   Mm1
....................uccaggcacugucguucCgucg......................................................................   36     1   Mm1
....................uUcaggcacugucguucugucg......................................................................   18     1   Mm1
....................uccaggcacugucguuUugucg......................................................................   11     1   Mm1
....................ucGaggcacugucguucugucg......................................................................   1      1   Mm1
....................uccaggcacuCucguucugucg......................................................................   5      1   Mm1
....................uccaggcGcugucguucugucg......................................................................   1      1   Mm1
....................uccaggcacugucguucAgucg......................................................................   43     1   Mm1
....................ucAaggcacugucguucugucg......................................................................   2      1   Mm1
....................Cccaggcacugucguucugucg......................................................................   2      1   Mm1
....................uccaUgcacugucguucugucg......................................................................   1      1   Mm1
....................uccGggcacugucguucugucg......................................................................   1      1   Mm1
....................uccaggcacugucgAucugucg......................................................................   1      1   Mm1
....................uccaggcacugucguucugucgA.....................................................................   252    1   Mm1
....................uccaggcacugucguuAugucgc.....................................................................   1      1   Mm1
....................uccaggcacugucguucGgucgc.....................................................................   2      1   Mm1
....................uccaggcacugucguucugucgc.....................................................................   470    0   Mm1
....................uccaggcacugucguucCgucgc.....................................................................   2      1   Mm1
....................uccaggcacugucguucugucgU.....................................................................   413    1   Mm1
....................uccagAcacugucguucugucgc.....................................................................   1      1   Mm1
....................uccaggcacuCucguucugucgc.....................................................................   1      1   Mm1
....................Gccaggcacugucguucugucgc.....................................................................   1      1   Mm1
....................uccaggcacugucguuGugucgc.....................................................................   2      1   Mm1
....................uccaggcacugucguucAgucgc.....................................................................   5      1   Mm1
....................uccaggcacugucguucuguAgc.....................................................................   1      1   Mm1
....................uccaggcacugucguucugucgUu....................................................................   140    1   Mm1
....................uccaggcacugucguucugucgcu....................................................................   81     0   Mm1
....................uccaggcacugucguucugucgAu....................................................................   11     1   Mm1
....................Nccaggcacugucguucugucgcu....................................................................   2      1   Mm1
....................uccaggcacugucguucugucgcA....................................................................   69     1   Mm1
....................uccaggcacugucguucugucgcuA...................................................................   1      1   Mm1
....................uccaggcacugucguucugucgcuU...................................................................   34     1   Mm1
....................uccaggcacugucguucugucgcuguaaucguaauguggu....................................................   1      0   Mm1
......................caggcacugucguucuUucg......................................................................   1      1   Mm1
......................caggcacugucguucugucg......................................................................   14     0   Mm1
......................caggcacugucguucugCcg......................................................................   1      1   Mm1
...............................................aucguaaugugguagaaugaccuugccugauc.................................   1      0   Mm1
..........................................................guagaaugaccuugccugau..................................   3      0   Mm1
..........................................................guagaaugaccuugccuUauc.................................   1      1   Mm1
..........................................................guagaaugaccuugccugauc.................................   33     0   Mm1
..........................................................guagaaugacAuugccugaucc................................   1      1   Mm1
..........................................................guagaaugaccuugccuUaucc................................   3      1   Mm1
..........................................................guagaaugaccuugccugaucc................................   200    0   Mm1
..........................................................guagaaugaccuugccugaucA................................   1      1   Mm1
..........................................................guagaaugaccuCgccugaucc................................   1      1   Mm1
..........................................................guagaaugaccuugccAgaucc................................   1      1   Mm1
..........................................................guagaaugacGuugccugaucc................................   1      1   Mm1
..........................................................guagUaugaccuugccugaucc................................   1      1   Mm1
..........................................................guagaaugaccuugccugauccU...............................   112    1   Mm1
...........................................................uagaaugaccuugccugaucc................................   5      0   Mm1

.Aauugacggacucguggg.............................................................................................   1      1   Mm2
....................uccaggcacugucguuAu..........................................................................   1      1   Mm2
```

```
                        Mature                                Star
ucauugacggacucgugggguuccaggcacugucguucugucgcuguaaucguaauguguguagaaugaccuugccugaucccaagggaacaucaacaucuggacacuugaac
....................uccaggcacugucguucA..........................................................................    1     1    Mm2
....................uccaggcacugucguuUu..........................................................................    1     1    Mm2
....................uccaggcacugucguucu..........................................................................    24    0    Mm2
....................uccaggcacugucguucuU.........................................................................    5     1    Mm2
....................uccaggcacugucguucuC.........................................................................    1     1    Mm2
....................uccaggcacugucguucug.........................................................................    27    0    Mm2
....................uccaggcacugucguucugu........................................................................    129   0    Mm2
....................uccaggcacugucguucGgu........................................................................    1     1    Mm2
....................uccaggcacugucguucAgu........................................................................    1     1    Mm2
....................uccaggcacugucguucCgu........................................................................    2     1    Mm2
....................uccaggcacuUucguucuguc.......................................................................    1     1    Mm2
....................uccaggcacugucguucuguA.......................................................................    6     1    Mm2
....................uccaggcacugucguucuguG.......................................................................    1     1    Mm2
....................uccaggcacugucguucuguU.......................................................................    4     1    Mm2
....................uccaggcacugucguuAuguc.......................................................................    1     1    Mm2
....................uccaggUacugucguucuguc.......................................................................    1     1    Mm2
....................uccaggcacugucguucCguc.......................................................................    3     1    Mm2
....................uccaggcacuAucguucuguc.......................................................................    1     1    Mm2
....................Cccaggcacugucguucuguc.......................................................................    1     1    Mm2
....................uccaggcacugucguucAguc.......................................................................    5     1    Mm2
....................uccaggcaUugucguucuguc.......................................................................    1     1    Mm2
....................uccaggcacugucguucuguc.......................................................................    564   0    Mm2
....................uccaggcacugucguucGguc.......................................................................    4     1    Mm2
....................Nccaggcacugucguucugucg......................................................................    1     1    Mm2
....................uccaggcacugucgAucugucg......................................................................    1     1    Mm2
....................ucAaggcacugucguucugucg......................................................................    1     1    Mm2
....................uccaggcacugucguucuguAg......................................................................    3     1    Mm2
....................uccagCcacugucguucugucg......................................................................    1     1    Mm2
....................uccaggcacugucguucCgucg......................................................................    37    1    Mm2
....................uccaggcacugucguuUugucg......................................................................    6     1    Mm2
....................uAcaggcacugucguucugucg......................................................................    3     1    Mm2
....................uccaggcacugucguucuAucg......................................................................    2     1    Mm2
....................uccaggcGcugucguucugucg......................................................................    4     1    Mm2
....................uccaggcaGugucguucugucg......................................................................    1     1    Mm2
....................uccaggcacuguAguucugucg......................................................................    1     1    Mm2
....................uccaggcacuCucguucugucg......................................................................    7     1    Mm2
....................uccaggcacugucguucugucA......................................................................    14    1    Mm2
....................uccaggcacGgucguucugucg......................................................................    1     1    Mm2
....................uccaggcacuUucguucugucg......................................................................    4     1    Mm2
....................uccaggUacugucguucugucg......................................................................    3     1    Mm2
....................uccaggcacugucguuGugucg......................................................................    9     1    Mm2
....................ucGaggcacugucguucugucg......................................................................    2     1    Mm2
....................Cccaggcacugucguucugucg......................................................................    2     1    Mm2
....................uGcaggcacugucguucugucg......................................................................    3     1    Mm2
....................uccaggcacugucguucugucU......................................................................    105   1    Mm2
....................uccaggcacugucguucugCcg......................................................................    1     1    Mm2
....................uccaggcacugucAuucugucg......................................................................    2     1    Mm2
....................uccaggcacugucguucGgucg......................................................................    27    1    Mm2
....................uccGggcacugucguucugucg......................................................................    3     1    Mm2
....................uccaggcacugucguuAugucg......................................................................    38    1    Mm2
....................Gccaggcacugucguucugucg......................................................................    1     1    Mm2
....................uccaggcacugucguucugucC......................................................................    1     1    Mm2
....................uccaggcacugucguucugAcg......................................................................    2     1    Mm2
....................uccaggcUcugucguucugucg......................................................................    3     1    Mm2
....................uccaggcacugucUuucugucg......................................................................    1     1    Mm2
....................uccaggcacuguUguucugucg......................................................................    1     1    Mm2
....................uccagUcacugucguucugucg......................................................................    5     1    Mm2
....................uccaggcacugucguucuguUg......................................................................    2     1    Mm2
....................uccaggcacugucguucAgucg......................................................................    35    1    Mm2
....................uccaggcCcugucguucugucg......................................................................    1     1    Mm2
....................ucUaggcacugucguucugucg......................................................................    2     1    Mm2
....................uccaggcacugucguucugucg......................................................................    6199  0    Mm2
....................uccaggcacuAucguucugucg......................................................................    9     1    Mm2
....................uccaggcaUugucguucugucg......................................................................    3     1    Mm2
....................uccaggcacugucguuGugucgc.....................................................................    1     1    Mm2
....................uccaggcacugucguucugucUc.....................................................................    1     1    Mm2
....................uccaggcacugucguucCgucgc.....................................................................    4     1    Mm2
....................uccaggcacugucguucugucgU.....................................................................    291   1    Mm2
....................uccaggcacugucguucugCcgc.....................................................................    1     1    Mm2
....................uccUggcacugucguucugucgc.....................................................................    1     1    Mm2
```

Mature **Star**

```
ucauugacggacucgugggu uccaggcacugucguucugucg cuguaaucguaaugug guagaaugaccuugccugaucc ccaagggaacaucaacaucuggacacuugaac
....................uccaggcacugucguuAugucgc.....................................................................   3     1   Mm2
....................uccaggcacugucguucAgucgc.....................................................................   1     1   Mm2
....................uccaggcacuCucguucugucgc.....................................................................   2     1   Mm2
....................uccaggcacugucguucugucgc.....................................................................   570   0   Mm2
....................uccaggcacugucguucugucgG.....................................................................   9     1   Mm2
....................uccaggcacugucguuUugucgc.....................................................................   1     1   Mm2
....................uccaggcacugucguucugucgA.....................................................................   145   1   Mm2
....................uccaggcacugucguucGgucgc.....................................................................   1     1   Mm2
....................uccaggcacugucguucugucgAu....................................................................   1     1   Mm2
....................uccaggcacuguUguucugucgcu....................................................................   1     1   Mm2
....................uccaggcacugucguucugucgcu....................................................................   120   0   Mm2
....................uccaggcacugucguucugucgUu....................................................................   78    1   Mm2
....................uccaggcacugucguucugucgcG....................................................................   9     1   Mm2
....................uccaggcacugucguucugucgcA....................................................................   24    1   Mm2
....................uccaggcacugucguucAgucgcu....................................................................   1     1   Mm2
....................ucGaggcacugucguucugucgcu....................................................................   1     1   Mm2
....................uccaggcacugucguucugucgcuU...................................................................   22    1   Mm2
....................uccaggcacugucguucugucgcuguU.................................................................   1     1   Mm2
....................uccaggcacugucguucugucgcuguaau...............................................................   1     0   Mm2
.....................ccaggcacugucguucugucg......................................................................   2     0   Mm2
.....................Ucaggcacugucguucugucg......................................................................   1     1   Mm2
.....................ccaggcacugucguucugucgc.....................................................................   2     0   Mm2
......................caggcacugucguucuguc.......................................................................   2     0   Mm2
......................caggcacugucguucugucg......................................................................   21    0   Mm2
......................caggcacugucguucugAcg......................................................................   2     1   Mm2
......................caggAacugucguucugucg......................................................................   1     1   Mm2
......................caggcacugucguucugucgc.....................................................................   1     0   Mm2
......................caggcacugucguucugucgcA....................................................................   1     1   Mm2
.......................aggcacugucguucuguAg......................................................................   1     1   Mm2
.......................aggcacugucguucugucg......................................................................   1     0   Mm2
........................................cgcuguaaucguaaugugguagaaugaccuugccu.....................................   1     0   Mm2
..........................................cuguaaucguaaugugguagaaugacc...........................................   2     0   Mm2
..........................................cuguaaucguaaugugguagaaugaccuu.........................................   1     0   Mm2
...........................................uguaaucguaaugugguagaaug..............................................   1     0   Mm2
...........................................uguaaucguaaugugguagaaugacc...........................................   1     0   Mm2
...........................................uguaaucguaaugugguagaaugaccuug........................................   2     0   Mm2
...........................................uguaaucguaaugugguagaaugaccuugccu.....................................   1     0   Mm2
...........................................uguaaucguaaugugguagaaugaccuugccugauc.................................   1     0   Mm2
.........................................................gguagaaugaccuugccugaucc................................   1     0   Mm2
..........................................................guagaaugaccuugccugU...................................   1     1   Mm2
..........................................................guagaaugaccuugccuga...................................   14    0   Mm2
..........................................................guagaaugaUcuugccugau..................................   1     1   Mm2
..........................................................guagaaugaccuugccugau..................................   44    0   Mm2
..........................................................guagaaugaccuugccugauU.................................   7     1   Mm2
..........................................................guaCaaugaccuugccugauc.................................   1     1   Mm2
..........................................................guagaaugUccuugccugauc.................................   1     1   Mm2
..........................................................guagaaugaccuugAcugauc.................................   1     1   Mm2
..........................................................guagaaugaccuugccuUauc.................................   2     1   Mm2
..........................................................Uuagaaugaccuugccugauc.................................   1     1   Mm2
..........................................................guagaaugaccuugccuCauc.................................   1     1   Mm2
..........................................................guagaaugaccuugccuAauc.................................   1     1   Mm2
..........................................................guagaaugaccuugccugauc.................................   261   0   Mm2
..........................................................guagaaugaccuugccugauA.................................   1     1   Mm2
..........................................................guagaauCaccuugccugaucc................................   1     1   Mm2
..........................................................guagaaugacGuugccugaucc................................   5     1   Mm2
..........................................................guagaaugaccuugccugGucc................................   1     1   Mm2
..........................................................guagaaugaccuuAccugaucc................................   1     1   Mm2
..........................................................guagGaugaccuugccugaucc................................   1     1   Mm2
..........................................................guagaauUaccuugccugaucc................................   1     1   Mm2
..........................................................guagaaAgaccuugccugaucc................................   2     1   Mm2
..........................................................guagaaugaccuugccugauAc................................   1     1   Mm2
..........................................................guagCaugaccuugccugaucc................................   1     1   Mm2
..........................................................guaUaaugaccuugccugaucc................................   2     1   Mm2
..........................................................guagaaugGccuugccugaucc................................   2     1   Mm2
..........................................................guagaaugaccuugccCgaucc................................   8     1   Mm2
..........................................................guagUaugaccuugccugaucc................................   1     1   Mm2
..........................................................guGgaaugaccuugccugaucc................................   1     1   Mm2
..........................................................guagaaugaccuugccuAaucc................................   16    1   Mm2
..........................................................Auagaaugaccuugccugaucc................................   1     1   Mm2
..........................................................guagaaugaccuugccugauUc................................   1     1   Mm2
```

```
                    Mature                                  Star
ucauugacggacucguggguuccaggcacugucguucugucgcuguaaucguaauguguagaaugaccuugccugauccccaagggaacaucaacaucuggacacuugaac
..........................................................guagaaugaccuugccGgaucc................................    9      1     Mm2
..........................................................guagaaugaccuugccugaucc................................    3318   0     Mm2
..........................................................guagaaugaccuugccugaucU................................    44     1     Mm2
..........................................................guagaaugacAuugccugaucc................................    12     1     Mm2
..........................................................guagaGugaccuugccugaucc................................    1      1     Mm2
..........................................................guagaaugaccuugccAgaucc................................    9      1     Mm2
..........................................................guagaaugaccuugccuCaucc................................    12     1     Mm2
..........................................................guagaaugaccuugccuUaucc................................    18     1     Mm2
..........................................................guagaaugCccuugccugaucc................................    6      1     Mm2
..........................................................guagaaugacUuugccugaucc................................    2      1     Mm2
..........................................................guagaaugaccuugccugaucA................................    2      1     Mm2
..........................................................guagaaugUccuugccugaucc................................    2      1     Mm2
..........................................................guaAaaugaccuugccugaucc................................    1      1     Mm2
..........................................................guagaaugaccAugccugaucc................................    1      1     Mm2
..........................................................Uuagaaugaccuugccugaucc................................    1      1     Mm2
..........................................................guagaaugaccuuUccugaucc................................    2      1     Mm2
..........................................................guagaaugaccuugAcugaucc................................    1      1     Mm2
..........................................................guagaaugaccCugccugaucc................................    1      1     Mm2
..........................................................guagaaugaccuugccugaAcc................................    1      1     Mm2
..........................................................guagaaugaccuugccugauccG...............................    19     1     Mm2
..........................................................guagaaugaccuugccugauccA...............................    114    1     Mm2
..........................................................guagaaugaccuugccugauccc...............................    1      0     Mm2
..........................................................guagaaugaccuugccugauccU...............................    540    1     Mm2
..........................................................guagaaugaccuugccugauccUc..............................    2      1     Mm2
...........................................................uagaaugaccuugccugaucc................................    2      0     Mm2
```
